# Association between physical activity and falls among older adults in rural China: are there gender and age related differences?

**DOI:** 10.1186/s12889-022-12773-1

**Published:** 2022-02-19

**Authors:** Yemin Yuan, Jie Li, Peipei Fu, Zhengyue Jing, Yi Wang, Chengchao Zhou

**Affiliations:** 1 Centre for Health Management and Policy Research, School of Public Health, Cheeloo College of Medicine, Shandong University, Jinan, 250012 China; 2 Department of Epidemiology, School of Public Health, Cheeloo College of Medicine, Shandong University, Jinan, 250012 China; 3 School of Health Policy and Management, Nanjing Medical University, Nanjing, 211166 China; 4 NHC Key Laboratory of Health Economics and Policy Research, Shandong University, Jinan, 250012 China

**Keywords:** Physical activity, Moderate-to-vigorous physical activity, Falls, Older adults, Gender, Age, Differences

## Abstract

**Background:**

The relationship between physical activity (PA) and falls among older adults is inconsistent, and little is known about the gender-specific association between falls and PA. Moreover, age may modify this relationship. This study aimed to test the association between PA and falls and to investigate the gender and age differences in the association among rural older adults.

**Methods:**

This cross-sectional data were derived from the baseline survey of Shandong Rural Elderly Health Cohort (SREHC). In total, 3,242 rural older adults aged 60 years and above were included in the analysis. PA was measured by the International Physical Activity Questionnaire Short Form (IPAQ-S). PA levels were classified as low, moderate, elevated and high according to quartiles. Volume of moderate-to-vigorous physical activity (MVPA) was categorized into low, moderate, elevated, and high level based on global recommendations. Information on falls was determined from in-person interviews. Falling was defined to participants as ending up on the floor or ground because they were unable to stop themselves. Logistic regression analysis was employed to explore the association between falls and PA.

**Results:**

Of 3,242 rural older adults, the incidence of falls was 13.1%. In older adults, high levels of PA [odds ratio (OR) = 0.65, 95% confidence interval (CI): 0.47–0.90] or MVPA (OR = 0.68, 95% CI: 0.50–0.94) were related to falls. Moderate (OR = 4.84, 95% CI: 1.68–13.94) or high (OR = 0.54, 95% CI: 0.30–0.99) levels of MVPA were associated with falls in older men. But elevated levels of PA were associated with falls (OR = 0.60, 95% CI: 0.42–0.87) in older women. Among older people younger than 75 years, elevated (OR = 0.54, 95% CI: 0.37–0.79) or high (OR = 0.68, 95% CI: 0.48–0.98) levels of PA were associated with falls.

**Conclusions:**

Among Chinese rural older adults, PA and MVPA are associated with falls, and there are gender and age differences. To prevent falls, measures need to account for individuals’ gender and age to encourage rural older adults to participate more actively in PA. We will conduct longitudinal studies to clarify the causal relationship between PA and fall.

**Supplementary Information:**

The online version contains supplementary material available at 10.1186/s12889-022-12773-1.

## Background

Falls are a leading cause of serious injuries, disabilities and death among older adults [1, 2]. According to the World Health Organization's estimation, each year an estimated 684,000 individuals die from falls globally, and adults older than 60 years of age suffer the greatest number of fatal falls [2]. Data from National Injury Monitoring System (2014) in China found among people aged 60 and over, there were 77,779 accidental injuries, of which 52.8% were caused by falls [3]. Falls and affiliated injuries place enormous pressures on national healthcare system and increase medical cost [4]. Early identification of risk factors of falls for older adults is important for preventing falls.

Physical activity (PA) is important for preserving physical function and mobility, which can then delay the onset of major disability [5]. PA, as a single intervention, is the most commonly tested fall prevention intervention and previous studies have shown PA can prevents falls [6]. Some studies found that low or high levels of PA were associated with falls in older adults [7, 8], indicating that there was a U-shaped relationship between PA and falls. In contrast, other studies reported that high levels of PA could reduce the risk of falls [9, 10]. A recent ten-year population-based longitudinal study in Australia found that older adults who increased their own moderate-to-vigorous physical activity (MVPA) further reduced the risk of falls [11]. Consistent evidence shows that low levels of activity are associated with falls among older adults. However, it remains controversial that whether high levels of activity would eventually increase falls.

A population-based study in the United States found that older women were more likely than older men to report falls and seek related medical care [12]. Previous studies have found that older women had a higher incidence of falls than older men [4, 13, 14]. Lower mid-calf muscle was independently associated with a higher likelihood of falls in both women and men [15]. A follow-up study in the United States found that older men and women with frequent falls had different PA patterns [16]. Although several studies have demonstrated the gender differences in falls and PA separately, it is unclear whether there are gender differences in the association between PA and falls. In addition, the rate of fall-related injuries increases with age [17]. In particular, adults aged 75 and older had more age-related loss of physical function [18]. However, research evidence suggested that PA programs improved physical function and reduced risk of age-related loss of physical function in the general aging population [19]. Thus, age may also modify the relationship between PA and falls.

Studies found the incidence of falls and PA among older adults in rural China was higher than that of older adults in urban China [20, 21]. Therefore, the current study aims to examine the relationship between PA and falls in Chinese rural older adults, and to explore the gender and age differences in the relationship between PA and falls.

## Methods

### Study setting and participants

This study used the cross-sectional data from the baseline survey of Shandong Rural Elderly Health Cohort (SREHC), which was conducted from May to June 2019 in Shandong province, China. Shandong is not only the second most populous provinces of China with 107 million people in 2018, but also with the largest number of aging population. People aged 60 and over accounted for 22.3% of the total population in 2018. A multistage stratified cluster sampling method was used to select participants. Firstly, according to the Gross Domestic Product (GDP) per capita in Shandong in 2018, we selected three rural counties (Rushan in Weihai, Qufu in Jining, and Laoling in Dezhou) as study sites. Secondly, five townships were randomly selected from each rural county. Thirdly, we randomly selected four villages from each of the selected townships. Finally, people aged or over 60 years old were randomly selected from each sample village. Inclusion criteria were (1) permanent residents who lived in the village for over 6 months in the past year, and (2) aged 60 years old and above. Exclusion criterion were (1) respondents who had an inability to complete the interview or communicate with others, and (2) with a history of dementia by further asking the village doctors about the physical condition of the interviewees. We also excluded the elderly who were unable to walk independently. All the respondents were interviewed face-to-face using a structured questionnaire by trained interviewers with medical knowledge. The completed questionnaires were carefully checked by the supervisors each day. In total, 3,600 individuals were recruited and 3,243 completed the whole survey, with a response rate of 90.1%. Of the 3,243 respondents, one respondent was excluded from analysis due to the lack of data in cognitive survey. Finally, a total of 3,242 rural older adults were included in the analysis.

We used the following formula to calculate the sample size [22]:${\text{N}} = \frac{{u_{\alpha /2}^{2} \pi (1 - \pi )}}{{\delta^{2} }}$(*π*: expected prevalence). Previous research showed that the prevalence of falls among Chinese rural older people ranged from 13.5% to 36.4% [23, 24], so in this study, $\pi$ = 13%, $u_{\alpha /2}$ = 1.96, $\delta$ = 0.1, $\pi$ = 0.013. We found that the required sample size was 2,571. The total sample size obtained in our study is 3,242, which has reached the required sample size.

### Measures

#### Falls

Falls were determined from in-person interviews. Falling was defined to participants as ending up on the floor or ground because they were unable to stop themselves. At the time of the interview, participants were asked “Have you had ever fallen on the floor or ground during the past 12 months?” (yes, no) and “If yes, how many falls have you had in the past 12 months?” [25]. Falls status was categorized as having no falls versus falls.

#### Physical activity

Physical activity (PA) was measured by the Chinese version of International Physical Activity Questionnaire Short Form (IPAQ-S). The IPAQ-S contains seven questions, six of which are about PA and the seventh is sedentary time. More details were described in Additional file 1: Appendix 1. The reliability and validity of the IPAQ-S have been confirmed in China [26]. IPAQ-S includes vigorous physical activity (VPA), moderate physical activity (MPV) and walking. According to recognized standards, a metabolic equivalent value (MET) was assigned to each type of activity [27], where 1 MET was a resting metabolic rate obtained during quiet sitting. MET value was 8 for VPA, 4 for MPA, and 3.3 for walking. The individuals participating in some intensity PA levels each week were: the MET value corresponding to this PA × frequency per week (days/week) × daily time (minutes/days). The sum of the three levels of PA is the overall levels of PA [28]. The levels of PA were divided into quartiles (low level: < 694 MET, moderate level: 694–2,772 MET, elevated level: 2,773–4,158 MET, and high level: > 4,158 MET). We recalculated the quartiles of PA levels in each subgroup (male: low level: < 694 MET, moderate level: 694–2,772 MET, elevated level: 2,773–4,692 MET, and high level: > 4,692 MET; female: low level: < 694 MET, moderate level: 694–2,772 MET, elevated level: 2,773–4,158 MET, and high level: > 4,158 MET; < 75 years: low level: < 694 MET, moderate level: 694–2,772 MET, elevated level: 2,773–4,158 MET, and high level: > 4,158 MET; and ≥ 75 years: low level: < 578 MET, moderate level: 578–1,386 MET, elevated level: 1,386–4157 MET, and high level: > 4,157 MET). Concerning moderate-to-vigorous physical activity (MVPA), the value was computed by multiplying the volume of VPA and MPA by an assigned MET. Since the lower and higher cut-off points for the sufficient MVPA are 150 min/week MPA and 300 min/week VPA [29], which were equal to 600 MET and 2400 MET, respectively. Therefore, the volume of MVPA was categorized into low level (0 MET), moderate level (1–599 MET), elevated level (600–2399 MET), and high level (≥ 2400 MET).

#### Covariates

In this study, covariates included sociodemographic, health behavior and health status variables. Sociodemographic variables included gender, age, education, and household income. We measured age, education, and household income by asking participants about their year of birth, their highest level of education, and their total household income in 2018. Household income was divided into quartiles (quartile 1: ≤ 882 $, quartile 2: > 882 $ & ≤ 1,730 $, quartile 3: > 1,730 $ & ≤ 4,217 $, quartile 4: > 4,217 $. Average exchange rate in 2018: 1 ¥ = 6.612 $). of household income in each subgroup (male: quartile 1: ≤ 944 $, quartile 2: > 944 $ & ≤ 1,730 $, quartile 3: > 1,730 $ & ≤ 4,008 $, quartile 4: > 4,008 $; female: quartile 1: ≤ 817 $, quartile 2: > 817 $ & ≤ 1,730 $, quartile 3: > 1,730 $ & ≤ 4,338 $, quartile 4: > 4,338 $; < 75 years: quartile 1: ≤ 1,028 $, quartile 2: > 1,028 $ & ≤ 2,024 $, quartile 3: > 2,024 $ & ≤ 4,755 $, quartile 4: > 4,755 $; and ≥ 75 years: quartile 1: ≤ 580 $, quartile 2: > 580 $ & ≤ 970 $, quartile 3: > 970 $ & ≤ 2,099 $, quartile 4: > 2,099 $). Health behavior variables included alcohol consumption. Alcohol consumption was measured by asking about the frequency of drinking over the previous 12 months. Alcohol consumption refers to drinking more than once a week for more than 6 months [30]. Health status variables included body mass index (BMI, calculated from most recent height and weight), living alone, number of chronic disease, activities of daily living (ADL), psychological distress (PD), and Mini-Mental State Examination (MMSE). Height (in centimeter) was self-reported and weight (in kilogram) was measured by the interviewers. Living alone was identified by the question: “Who do you live with now?”. The answers were the following five types: living alone; living only with spouse; living only with adult children; living both with spouse and adult children; and living with people other than the above. Chronic disease was measured by the questions: “Have you ever been diagnosed with a chronic disease by a physician?”. If the answer was “yes”, participants were further asked “How many chronic diseases have you ever been diagnosed?”, which was validated by the chronic disease case management system. ADL were measured by the Physical Self-Maintenance Scale (PSMS) and the Instrumental Activity of Daily Living Scale (IADL) [31]. The 14-item ADL score ranges from 14 to 56, with higher scores indicating the worse ability of daily living activities. PD was measured by the Chinese version of the 10-item Kessler Psychological Distress Scale (K10) [32]. The total score ranges from 10 to 50. The higher the score, the worse the mental health status. MMSE was measured using the 30-item Chinese version of the Mini-Mental State Examination [33]. The maximum score is 30 points, with the higher scores indicating a better cognitive function.

### Statistical analysis

Data were analyzed using SPSS version 24.0. Descriptive statistics were used to describe characteristics of participants based on gender with mean (standard deviation) or frequency (percentage). Pearson’s chi-square test for categorical variables and Student’s t test for continuous variables were used to compare individual characteristics among male and female adults. Associations between PA and MVPA with falls were assessed by binary logistic regression. Logistic regression was used to examine the interactions of gender, age and PA with MVPA. Associations between PA and MVPA with falls stratified by gender and age were also assessed by binary logistic regression. The odds ratio (OR) and 95% confidence interval (CI) were presented as measures of effect. In model 1, none covariates were included. Based on previous research [34, 35], we found that age, gender, education and other confounding factors may also be associated with falls among older adults. Therefore, model 2 was based on model 1, with additional adjusting for these covariates. When we conducted the analysis in each subgroup, we recalculated the quartiles of continuous values (levels of PA and household income) in each subgroup (male, female, < 75 years, and ≥ 75 years). We also tested the curve relationship between PA and falls using curvilinear regression (see Additional file 2: Appendix 2). The statistically significant threshold was set at a two-sided and *p*-value < 0.05.

## Results

### Characteristics

Participants’ characteristics by gender are shown in Table 1. Among 3,242 participants, age ranged from 60 to 100 (Mean = 70.14). About two-thirds of the participants were female. Compared with older men participants, older women participants tended to be younger. In terms of lifestyle behaviors, older men were more likely to drink than older women. For health status, older women were more likely to live alone, have more chronic diseases, and have high ADL and PD scores. But older men had higher MMSE scores than older women.

**Table 1:** Participants’ characteristics by gender

Characteristics	N (%)	Male, n (%)	Female, n (%)	*p-*valve
**Total**	3,242(100.00)	1,181(36.43)	2,061(63.57)	
**Age (years)**, Mean (SD)	70.14(6.17)	70.58(6.25)	69.89(6.11)	0.002
** < 75**	2,499(77.08)	883(74.77)	1,616(78.41)	0.018
** ≥ 75**	743(22.92)	298(25.23)	445(21.59)	
**Education**				< 0.001
Illiteracy	1,353(41.73)	266(22.52)	1,087(52.74)	
Primary school	1,257(38.77)	539(45.64)	718(34.84)	
Junior high school and above	632(19.50)	376(31.84)	256(12.42)	
**Household income**^**a**^				< 0.001
Quartile 1	816(25.17)	253(21.42)	563(27.32)	
Quartile 2	803(24.77)	337(28.54)	466(22.61)	
Quartile 3	809(24.95)	307(25.99)	502(24.36)	
Quartile 4	814(25.11)	284(24.05)	530(25.72)	
**Alcohol consumption**				< 0.001
No	2,320(71.56)	438(37.09)	1,882(91.31)	
Yes	922(28.44)	743(62.91)	179(8.69)	
**BMI (kg/m**^**2**^**)**, Mean (SD)	24.31(3.92)	24.24(3.79)	24.35(3.99)	0.443
**Living alone**				0.020
No	2,624(80.94)	981(83.07)	1,643(79.72)	
Yes	618(19.06)	200(16.93)	418(20.28)	
**Number of chronic disease**				< 0.001
0	896(27.64)	362(30.65)	534(25.91)	
1	1,205(37.17)	457(38.70)	748(36.29)	
≥ 2	1,141(36.19)	362(30.65)	779(37.80)	
**ADL (scores)**, Mean (SD)	16.99(4.37)	16.41(4.55)	17.33(4.22)	< 0.001
**PD (scores)**, Mean (SD)	16.60(7.46)	15.17(6.79)	17.42(7.70)	< 0.001
**MMSE (scores)**, Mean (SD)	22.91(5.11)	24.45(4.48)	22.03(5.24)	< 0.001
**PA**^**b**^, Mean (SD)	3,122.52(3157.60)	3220.17(3185.25)	2473.45(2887.96)	< 0.001
Low level	997(30.75)	336(28.45)	661(32.07)	< 0.001
Moderate level	905(27.91)	292(24.72)	613(29.74)	
Elevated level	679(20.94)	240(20.32)	439(21.30)	
High level	661(20.39)	313(26.50)	348(16.89)	
**MVPA**^**c**^, Mean (SD)	1691.50(4258.07)	1775.75(4370.81)	1131.60(3366.55)	0.004
Low level	2,310(71.25)	764(64.69)	1,546(75.01)	< 0.001
Moderate level	66(2.04)	21(1.78)	45(2.18)	
Elevated level	236(7.28)	84(7.11)	152(7.38)	
High level	630(19.43)	312(26.42)	318(15.43)	
**Falls**				< 0.001
No	2,818(86.92)	1,064(90.09)	1,754(85.10)	
Yes	424(13.08)	117(9.91)	307(14.90)	

*SD* Standard deviation, *BMI* Body mass index, *ADL* Activities of Daily Living, *PD* Psychological Distress, *MMSE* Mini-Mental State Examination, *PA* Physical activity, *MVPA* Moderate-to-vigorous physical activity

^**a**^Quartile 1: ≤ 882 $; quartile 2: > 882 $ & ≤ 1,730 $; quartile 3: > 1,730 $ & ≤ 4,217 $; quartile 4: > 4,217 $. Average exchange rate in 2018: 1 ¥ = 6.612 $

^**b**^Low level: < 694 MET; moderate level: 694–2,772 MET; elevated level: 2,773–4,158 MET; high level: > 4,158 MET

^**c**^Low level: 0 MET; moderate level: 1–599 MET; elevated level: 600–2,399 MET; high level: ≥ 2,400 MET

In addition, the proportion of older men engaged in low, moderate or elevated levels of PA was lower than that of older women. However, the proportion of older men participating in high levels of PA was significantly higher than that of older women. About 26.7% older adults performed sufficient amount of MVPA as recommended by the WHO. The distribution of MVPA in older men and women was similar to that of PA. The incidence of falls in the past 12 months was 13.1%. The distribution of falls is presented in Additional file 3: Appendix 3.

### Association between PA and MVPA with falls among older adults and subgroups

**Fig. 1:**
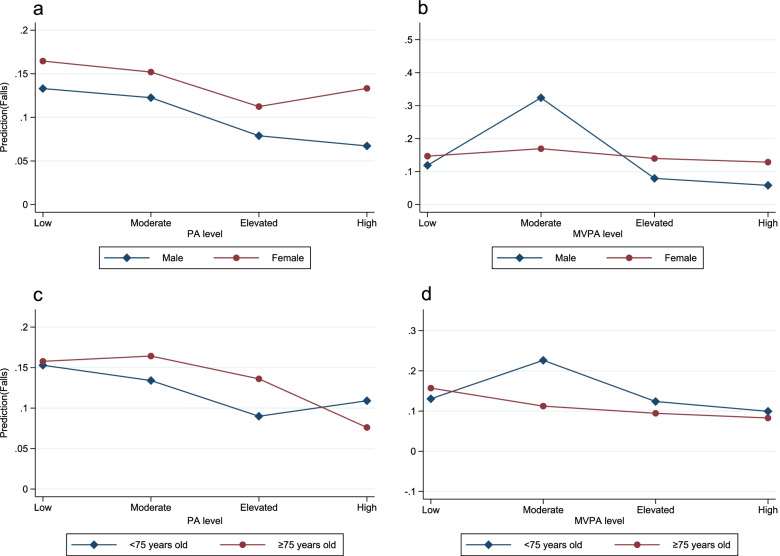
The interactions between gender, age and PA on falls among older adults PA, physical activity; MVPA, moderate-to-vigorous physical activity. **a** represents PA × gender. **b** represents MVPA × gender. **c** represents PA × age. **d** represents MVPA × age. Models were adjusted for gender, age, education, household income, alcohol consumption, BMI, living alone, number of chronic disease, ADL score, PD score and MMSE score

Table 2 presents the relationship between PA and falls in older adults. Elevated (OR = 0.61, 95% CI: 0.44–0.83, *p* = 0.002) and high (OR = 0.65, 95% CI: 0.47–0.90, *p* = 0.010) levels of PA were associated with falls. High levels of MVPA were related to falls (OR = 0.68, 95% CI: 0.50–0.94, *p* = 0.019). Figure 1 shows that the interactions of gender, age and PA with MVPA was decomposed by computing simple slopes of gender and age on different levels of PA and MVPA.

**Table 2:** Associations between PA and MVPA with falls among older adults

Variable	Model 1			Model 2		
	**OR**	**95% CI**	***p-*valve**	**OR**	**95% CI**	***p-*valve**
**PA**						
Low level	1.00			1.00		
Moderate level	0.80	0.62–1.03	0.082	0.90	0.70–1.17	0.448
Elevated level	0.51	0.38–0.70	< 0.001	0.61	0.44–0.83	0.002
High level	0.49	0.36–0.67	< 0.001	0.65	0.47–0.90	0.010
**MVPA**						
Low level	1.00			1.00		
Moderate level	1.62	0.89–2.95	0.118	1.74	0.93–3.25	0.084
Elevated level	0.78	0.51–1.18	0.232	0.85	0.55–1.30	0.447
High level	0.55	0.41–0.75	< 0.001	0.68	0.50–0.94	0.019

*PA* Physical activity, *MVPA* Moderate-to-vigorous physical activity, *OR* Ddds ratio, *CI* Confidence interval

Model 1 was unadjusted. Model 2 was adjusted for age, gender, education, household income, alcohol consumption, BMI, living alone, number of chronic disease, ADL score, PD score and MMSE score

Table 3 presents the relationship between PA and falls stratified by gender. Elevated levels of PA were associated with falls (OR = 0.60, 95% CI: 0.42–0.87, *p* = 0.007) only in older women. However, among older men, moderate levels of MVPA were associated with falls (OR = 4.84, 95% CI: 1.68–13.94, *p* = 0.003). High levels of MVPA were related to falls (OR = 0.54, 95% CI: 0.30–0.99, *p* = 0.049).

**Table 3:** Associations between PA and MVPA with falls stratified by gender

Variable	Male (*n* = 1,181)							Female (*n* = 2,061)					
	**Model 1**			**Model 2**			**Model 1**				**Model 2**		
	**OR**	**95% CI**	***p-*valve**	**OR**	**95% CI**	***p-*valve**	**OR**		**95% CI**	***p-*valve**	**OR**	**95% CI**	***p-*valve**
**PA**													
Low level	1.00			1.00			1.00				1.00		
Moderate level	0.79	0.49–1.27	0.328	1.10	0.66–1.84	0.722	0.80		0.59–1.07	0.128	0.87	0.64–1.18	0.368
Elevated level	0.50	0.26–0.82	0.013	0.72	0.39–1.31	0.275	0.55		0.38–0.78	0.001	0.60	0.42–0.87	0.007
High level	0.32	0.21–0.64	< 0.001	0.54	0.28–1.05	0.068	0.62		0.43–0.91	0.013	0.69	0.47–1.02	0.065
**MVPA**													
Low level	1.00			1.00			1.00				1.00		
Moderate level	3.00	1.13–7.92	0.027	4.84	1.68–13.94	0.003	1.18		0.54–2.56	0.681	1.17	0.53–2.60	0.694
Elevated level	0.58	0.24–1.36	0.208	0.73	0.30–1.80	0.493	0.88		0.54–1.41	0.578	0.87	0.53–1.43	0.587
High level	0.38	0.22–0.66	0.001	0.54	0.30–0.99	0.049	0.74		0.51–1.06	0.104	0.78	0.54–1.15	0.210

*PA* Physical activity, *MVPA* Moderate-to-vigorous physical activity, *OR* Odds ratio, *CI* Confidence interval. Model 1 was unadjusted. Model 2 was adjusted for age, education, household income, alcohol consumption, BMI, living alone, number of chronic disease, ADL score, PD score and MMSE score

Table 4 shows the relationship between PA and falls stratified by age. The association between PA or MVPA and falls was found only in older adults younger than 75 years old. Elevated (OR = 0.54, 95% CI: 0.37–0.79, *p* =  < 0.001) or high (OR = 0.68, 95% CI: 0.48–0.98, *p* = 0.037) levels of PA were negatively correlated with falls. Regression results based on single, multiple falls and continuous falls outcomes are shown in Additional file 4: Appendix 4.

**Table 4:** Associations between PA and MVPA with falls stratified by age

Variable	Aged 60–74 (*n* = 2,499)							Aged 75–100 (*n* = 743)					
	**Model 1**			**Model 2**			**Model 1**				**Model 2**		
	**OR**	**95% CI**	***p-*valve**	**OR**	**95% CI**	***p-*valve**	**OR**		**95% CI**	***p-*valve**	**OR**	**95% CI**	***p-*valve**
**PA**													
Low level	1.00			1.00			1.00				1.00		
Moderate level	0.74	0.55–0.99	0.042	0.86	0.63–1.16	0.320	1.06		0.61–1.83	0.834	1.09	0.62–1.93	0.758
Elevated level	0.45	0.31–0.64	< 0.001	0.54	0.37–0.79	0.001	1.07		0.61–1.88	0.813	1.13	0.63–2.04	0.685
High level	0.51	0.37–0.72	< 0.001	0.68	0.48–0.98	0.037	0.57		0.31–1.03	0.064	0.66	0.35–1.24	0.198
**MVPA**													
Low level	1.00			1.00			1.00				1.00		
Moderate level	1.97	1.04–3.73	0.037	1.95	1.00–3.82	0.051	0.52		0.07–4.07	0.529	0.73	0.09–5.89	0.767
Elevated level	0.85	0.54–1.34	0.475	0.96	0.60–1.53	0.863	0.57		0.20–1.64	0.300	0.54	0.18–1.60	0.268
High level	0.61	0.44–0.84	0.003	0.75	0.53–1.05	0.096	0.36		0.14–0.91	0.030	0.42	0.16–1.11	0.080

*PA* Physical activity, *MVPA* Moderate-to-vigorous physical activity, *OR* Odds ratio, *CI* Confidence interval. Model 1 was unadjusted. Model 2 was adjusted for gender, education, household income, alcohol consumption, BMI, living alone, number of chronic disease, ADL score, PD score and MMSE score

## Discussion

Of 3,242 rural older adults, we found the incidence of falls in the past 12 months before the survey was 13.1%. The incidence of falls in this study was lower than that of rural older adults (60 +) in Sichuan (36.4%) [24], Hubei (13.5%) [23], Ningxia (25.0%) [36], and Beijing (14.5%) [14], but higher than the 10.9% in rural older adults (65 +) in Shanghai [37]. The incidence of falls varies greatly in different regions of China. We also found gender differences in falls, with women more likely to be frequent fallers than men.

Although the general population in China is physically active, only 26.7% of rural older adults meet the WHO’s weekly MVPA recommendation to achieve health benefits. One possible explanation is that rapid urbanization often brings new ideas, cultures, and technologies, all of which facilitate an increasingly sedentary lifestyle [38] and reduce levels of PA in rural areas. Another possible reason is that there are no venues and facilities for activities in some rural areas. We also found that PA participation rates in older women were significantly lower than in older men. In Chinese rural areas, it is common that older women take care of the young grandchildren left at home, which may hinder their participation in PA.

An inverse association between PA and MVPA with falls in rural older adults was found. Consistent with our findings, several studies suggested an association between high levels of PA and fewer falls in older adults [11, 39]. Some other studies, in contrast, indicated that a U-shaped relationship between PA and falls, that was, both high and low levels of PA increased the risk of falls [7, 8]. PA may increase or decrease the risk of falls depending on the functional status of the older adults [39]. Older adults with poorer functional conditions are more likely to get tired or suffer from falls, which leads to lower levels of exercise. For older adults with high levels of PA, such activity may be beyond their abilities and increase the likelihood of falls. If physical function declined and was accompanied by high levels of PA, older adults might fall.

We found a sex-specific association between PA and MVPA with falls. Weekly participation in elevated levels of PA was negatively associated with falls in older women. But PA was not associated with falls in older men. Older men with moderate levels of MVPA each week were more likely to falls, and those with high levels of MVPA each week were less likely to falls. The study found that moderate MVPA was associated with more falls, which might be because that men misclassified themselves on PA and they truly were in low PA. There is lots of data on people overestimating their PA with self-reported questionnaires compared to an objective measure of PA [40]. This finding may also be related to the additional benefit of MVPA in improving muscle strength, balance and gait parameters. For instance, Pau et al. found that while both light and high PA help improve static balance, only the PA program characterized by superior levels of intensity was associated with an improvement in dynamic posture and all considered gait patterns [41]. However, a recent community survey in the United States showed that many falls occur in those with low activity/performance, but most falls occurred in older men (aged 71–91) with relatively high activity and/or reasonable performance characteristics [42]. This difference in high PA may be attributable to the lower overall participation of older men in MVPA in our study.

We found no correlation between MVPA and falls in older women. An Australian cohort study of 8,188 older women (aged 70–75) indicated that the highest levels of high intensity PA were associated with a reduced risk of falls and fractures, which was inconsistent with our findings[9]. This might be because that the relationship between PA and falls generally changes with age. About half of older women in the current study were under the age of 70. In the younger, high PA tends to be associated with more falls, whereas in the older, higher PA tends to be associated with fewer falls [43]. This might be due to the fundamental differences in daily quantities, patterns, and trends of PA between younger, healthier individuals and older, less-functionally intact persons.

A possible explanation for the gender differences in MVPA is the different division of family roles. Chinese traditional view holds that men manage affairs out of the family and women manage internal affairs. Older women in rural areas spend their time care giving activities (e.g., sweeping the house or caring for the baby or other family members), which could interfere with their continued MVPA participation. That is, they are more likely to be obligatorily active and not have the option to reduce activity in these domains. Usually, older men go out to do farm work. If they don’t have land, they will have more time to do exercise. This could be due to the above mentioned factor that older women are more committed to completing household chores than to engaging in PA. Sports and athletics have traditionally been restricted to and associated with males, masculinity, and the “manly domain”.

We also found the negative association between PA and falls occurred only in older adults younger than75 years. While PA offers many health benefits to older adults, people in their 60 s and 90 s may need very different volume of PA. Because older adults are over 75, they are accompanied by more loss of physical function [18]. High levels of PA could be risky.

This study has implications for practice and policy. The findings in this study implied for the public health service managers in rural communities to strengthen PA participation of older adults to prevent falls. In addition, rural community managers should build activity venues and facilities to encourage rural older adults to participate more actively in PA based on their gender. It is recommended to encourage older women or younger elderly to participate more and continue to participate in elevated levels of PA. It is recommended to encourage older men to participate in high levels of MVPA. At the same time, measures should be taken to prevent falls.

This study has several limitations. First, there may be recall bias of information about falls. Compared with more rigorous methods such as monthly falls diaries, our method of falls ascertainment (by annual questionnaire) might have underestimated the incidence of falls. Second, objective measures of PA should be included in future study. Thirdly, due to the cross-sectional data, we cannot determine the casual inference between PA and falls. Future longitudinal studies are needed to elucidate the causal association between PA and falls. Finally, the number of women account for two-thirds of our sample, which may prevent us from finding a true relationship between PA and falls in men. There may be two reasons for an imbalance between the sexes. First, in the latest China Statistical Yearbook, the proportion of men aged 60 and above is significantly lower than that of women [44]. Second, in rural China, men who are capable of working go out to do odd jobs or do farm work, and most of those who are at home are women. It was difficult to find older men during our interviews. In some other studies [45–47], similarly, the proportion of female elderly was significantly higher than that of male elderly.

## Conclusion

High PA is negatively correlated with falls, and there are gender and age differences in the relationship between PA and falls among Chinese rural older adults. Older men who participate in high levels of MVPA are less likely to fall. Older women or older people younger than 75 who participate in elevated levels of PA are less likely to fall. In rural areas, policy makers should encourage older adults to perform PA based on their gender and age to prevent falls.

## Supplementary Information

**Additional file 1:** **Appendix 1.** All items in the Chinese version of International Physical Activity Questionnaire Short Form (IPAQ-S)

**Additional file 2:** **Appendix 2.** Curvilinear regression.

**Additional file 3:** **Appendix 3.** Distribution of falls.

**Additional file 4:** **Appendix 4.** Regression results based on single and multiple falls outcomes.

## Data Availability

The datasets used and/or analysed during the current study are available from the corresponding author on reasonable request.

## Abbreviations

PA: Physical activity
MVPA: Moderate-to-vigorous physical activity
IPAQ-S: International Physical Activity Questionnaire Short Form
MET: Metabolic equivalent value
VPA: Vigorous physical activity
MPA: Moderate physical activity
ADL: Activities of daily living
PD: Psychological distress
MMSE: Mini-Mental State Examination
PSMS: Physical Self-Maintenance Scale
IADL: Instrumental Activity of Daily Living Scale
K10: 10-Item Kessler Psychological Distress Scale

## References

[1] Wit Kd, Merali Z, Kagoma YK, Mercier E (2020). Incidence of intracranial bleeding in seniors presenting to the emergency department after a fall: a systematic review. Injury.

[2] World Health Organization. Falls-Key facts (26 April 2021). https://www.who.int/news-room/fact-sheets/detail/falls

[3] Er Y, Duan L, Ye P, Jiang Y, Ji C, Deng X (2016). Epidemiological characteristics of fall in old population: results from National Injury Surveillance in China 2014. Chin J Epidemiol.

[4] Wang J, Chen Z, Song Y (2010). Falls in aged people of the Chinese mainland: epidemiology, risk factors and clinical strategies. Ageing Res Rev.

[5] Pahor M, Guralnik JM, Ambrosius WT, Blair S, Bonds DE, Church TS (2014). Effect of structured physical activity on prevention of major mobility disability in older adults: the LIFE study randomized clinical trial. JAMA.

[6] Gillespie LD, Robertson MC, Gillespie WJ, Sherrington C, Gates S, Clemson LM (2012). Interventions for preventing falls in older people living in the community. Cochrane Database Syst Rev.

[7] Gill TM, Pahor M, Guralnik JM, Mcdermott MM, King AC, Buford TW (2016). Effect of structured physical activity on prevention of serious fall injuries in adults aged 70–89: randomized clinical trial (LIFE Study). BMJ.

[8] Pereira CLN, Baptista F, Infante P (2014). Role of physical activity in the occurrence of falls and fall-related injuries in community-dwelling adults over 50 years old. Disabil Rehabil.

[9] Heesch KC, Byles JE, Brow WJ (2008). Prospective association between physical activity and falls in community-dwelling older women. J Epidemiol Community Health.

[10] Thibaud M, Bloch F, Tournoux-Facon C, Brèque C, Rigaud AS, Dugué BT (2012). Impact of physical activity and sedentary behaviour on fall risks in older people: a systematic review and meta-analysis of observational studies. Eur Rev Aging Phys Act.

[11] Balogun S, Winzenberg T, Wills K, Scott D, Jones G, Callisaya M (2018). Longitudinal associations between serum 25-hydroxyvitamin D, physical activity, knee pain and dysfunction and physiological falls risk in community-dwelling older adults. Exp Gerontol.

[12] Stevens JA, Ballesteros MF, Mack KA, Rudd RA, Decaro E, Adler G (2012). Gender differences in seeking care for falls in the aged medicare population. Am J Prev Med.

[13] Liu Z, Wang Q, Zhi T, Zhu Y, Wang Y, Wang Z (2016). Frailty index and its relation to falls and overnight hospitalizations in elderly Chinese people: a population-based study. J Nutr Health Aging.

[14] Zhang D, He Y, Liu M, Yang H, Wu L, Wang J (2016). Study on incidence and risk factors of fall in the elderly in a rural community in Beijing. Chin J Epidemiol.

[15] Scott D, Johansson J, McMillan LB, Ebeling PR, Nordstrom A, Nordstrom P (2019). Mid-calf skeletal muscle density and its associations with physical activity, bone health and incident 12-month falls in older adults: the Healthy Ageing Initiative. Bone.

[16] Stahl ST, Albert SM (2015). Gender differences in physical activity patterns among older adults who fall. Prev Med.

[17] Peel NM, Kassulke DJ, McClure RJ (2002). Population based study of hospitalised fall related injuries in older people. Inj Prev.

[18] DiPietro L, Campbell WW, Buchner DM, Erickson KI, Powell KE, Bloodgood B (2019). Physical Activity, Injurious Falls, and Physical Function in Aging: An Umbrella Review. Med Sci Sports Exerc.

[19] Physical Activity Guidelines Advisory Committee. Physical Activity Guidelines Advisory Committee Report, 2018. Washington, DC: US Department of Health and Human Services, 2008. https://health.gov/paguidelines/guidelines/report.aspx. Accessed December 24, 2021.

[20] Zhang L, Ding Z, Qiu L, Li A (2019). Falls and risk factors of falls for urban and rural community-dwelling older adults in China. BMC Geriatr.

[21] Zhu W, Chi A, Sun Y (2016). Physical activity among older Chinese adults living in urban and rural areas: a review. J Sport Health Sci.

[22] Sun Z, Sun ZQ, Xu YY (2002). Medical statistics (third edition). People’s Medical Publishing House(PMPH).

[23] Shi R, Wu L, Zhang T, Cao Z, Jiang X, Wang Y (2012). Elderly falling information and analysis of risk factors in Hubei Province. Acta Med Univ Sci Technol Huazhong.

[24] Zhang P, Zhao Z, Huang S (2019). Situation and influencing factors of elderly people falling in rural areas of Santai county of Sichuan province. Chin J Osteoporos Bone Miner Res.

[25] Ganz DA, Higashi T, Rubenstein LZ (2006). Monitoring falls in cohort studies of community-dwelling older people: effect of the recall interval. J Am Geriatr Soc.

[26] Macfarlane DJ, Lee CCY, Ho EYK, Chan KL, Chan DTS (2007). Reliability and validity of the Chinese version of IPAQ (short, last 7 days). J Sci Med Sport.

[27] Fan M, Lv J, He P (2014). Method for calculating the level of physical activity in the International Physical Activity Questionnaire. Chin J Epidemiol.

[28] Xu L, Jiang CQ, Lam TH, Zhang WS, Thomas GN, Cheng KK (2011). Dose-response relation between physical activity and cognitive function: Guangzhou Biobank Cohort Study. Ann Epidemiol.

[29] World Health Organization. Global recommendations on physical activity for health (1 January 2010). https://www.who.int/publications/i/item/9789241599979 26180873

[30] Chen W, Huang L, Han H, Lin Q, Hu Z, Zhang W (2018). Effect of preserved food intake combined with alcohol drinking and tobacco smoking on esophageal cancer: a case-control study. Chin J Public Health.

[31] Lawton MP, Brody EM (1969). Assessment of older people self-maintaining and Instrumental Activities of Daily Living. Gerontologist.

[32] Zhou C, Chu J, Wang T, Peng Q, He J, Zheng W (2008). Reliability and validity of 10-item Kessler scale (K10;Chinese version in evaluation of mental health status of Chinese population. Chin J Clin Psychol.

[33] Zhang T, Yan R, Chen Q, Ying X, Zhai Y, Li F (2018). Body mass index, waist-to-hip ratio and cognitive function among Chinese elderly: a cross-sectional study. BMJ Open.

[34] Lu Z, Lam FMH, Leung JCS, Kwok TCY (2020). The U-shaped relationship between levels of bouted activity and fall incidence in community-dwelling older adults: a prospective cohort study. J Gerontol A Biol Sci Med Sci.

[35] Lee D-CA, Lalor AF, Russell G, Stolwyk R, Brown T, Mcdermott F (2017). Understanding temporal relationships between depression, falls, and physical activity in a cohort of post-hospitalized older adults a breakthrough or a conundrum?. Int Psychogeriatr.

[36] He J. Incidence and risk factor analysis of falls among the elderly in urban and rural communities. Ningxia: Ningxia Medical University. China; 2015.

[37] Sun X (2019). Analysis of the risk factors of the elderly in the rural suburbs of a city. Guid Chin Med.

[38] Feng Z, Cramm JM, Nieboer AP (2019). A healthy diet and physical activity are important to promote healthy ageing among older Chinese people. J Int Med Res.

[39] Nastasi AJ, Ahuja A, Zipunnikov V, Simonsick EM, Ferrucci L, Schrack JA (2018). Objectively measured physical activity and falls in well-functioning older adults: findings from the baltimore longitudinal study of aging. Am J Phys Med Rehabil.

[40] Grande GD, Oliveira CB, Morelhão PK, Sherrington C, Tiedemann A, Pinto RZ (2020). Interventions promoting physical activity among older adults: a systematic review and meta-analysis. Gerontologist.

[41] Pau M, Leban B, Collu G, Migliaccio GM (2014). Effect of light and vigorous physical activity on balance and gait of older adults. Arch Gerontol Geriatr.

[42] Orwoll ES, Fino NF, Gill TM, Cauley JA, Strotmeyer ES, Ensrud KE (2019). The relationships between physical performance, activity levels, and falls in older men. J Gerontol A Biol Sci Med Sci.

[43] Cauley JA, Harrison SL, Cawthon PM, Ensrud KE, Danielson ME, Orwoll E (2013). Objective measures of physical activity, fractures and falls: the osteoporotic fractures in men study. J Am Geriatr Soc.

[44] National Bureau of Statistics (2020). China Statistical Yearbook 2020.

[45] Wu L, He Y, Jiang B, Liu M, Wang J, Yang S, et al. The association between the prevalence, treatment and control of hypertension and the risk of mild cognitive impairment in an elderly urban population in China. Hypertens Res. 2016;39:367–75. 10.1038/hr.2015.146. 10.1038/hr.2015.146 PMC4865472 26739869

[46] Zou Y, Zhu Q, Deng Y, Duan J, Pan L, Tu Q, et al. Vascular risk factors and mild cognitive impairment in the elderly population in Southwest China. Am J Alzheimers Dis Other Demen. 2014;29:242–7. 10.1177/1533317513517042. 10.1177/1533317513517042 PMC10852946 24375574

[47] Xu S, Xie B, Song M, Yu L, Wang L, An C, et al. High prevalence of mild cognitive impairment in the elderly: A community-based study in four cities of the Hebei Province, China[J]. Neuroepidemiology. 2014;42:123–30. 10.1159/000357374. 10.1159/000357374 24481120

