# Supplementary material for: Association between physical activity and falls among older adults in rural China: are there gender and age related differences?
Source: BMC Public Health. 2022 Feb 19;22:356. doi: 10.1186/s12889-022-12773-1 (PMC8858519; doi:10.1186/s12889-022-12773-1)
Supplement: Supplementary file 1 — Additional file 1: Appendix 1. All items in the Chinese version of International Physical Activity Questionnaire Short Form (IPAQ-S) [file 12889_2022_12773_MOESM1_ESM.doc]

**Appendix 1: All items in** **the Chinese version of International Physical Activity Questionnaire Short Form (****IPAQ-S).**

**Physical activity**

Physical activity (PA) was measured by the Chinese version of the International Physical Activity Questionnaire Short Form (IPAQ-S). The reliability and validity of the IPAQ-S have been confirmed in China [1,2]. The IPAQ-S asks participants to report activities performed for at least 10 minutes during the last 7 days. The scale reads as follows:

Think about all the vigorous activities that you did in the last 7 days. Vigorous physical activities refer to activities that take hard physical effort and make you breathe much harder than normal. Think only about those physical activities that you did for at least 10 minutes at a time.

**IPAQ-S**

1. During the last 7 days, on how many days did you do vigorous physical activities like heavy lifting (including farm work), running, swimming, playing football, playing basketball, playing tennis, jumping rope, aerobics, etc.?

_____ days per week

No vigorous physical activities Skip to question 3

2. How much time did you usually spend doing vigorous physical activities each day on one

of those days?

The average number of _____ hours _____ minutes per day

Think about all the moderate activities that you did in the last 7 days. Moderate activities refer to activities that take moderate physical effort and make you breathe somewhat harder than normal. Think only about those physical activities that you did for at least 10 minutes at a time.

3. During the last 7 days, on how many days did you do moderate physical activities like carrying light loads, bicycling at a regular pace, playing tai chi, joint exercises, fan dance, playing mulan boxing, playing table tennis, playing badminton, ballroom dancing, etc.? Do not include walking.

_____ days per week

No moderate physical activities Skip to question 5

4. How much time did you usually spend doing moderate physical activities each day on one

of those days?

The average number of _____ hours _____ minutes per day

Think about the time you spent walking in the last 7 days. This includes at work and at

home, walking to travel from place to place, and walking for exercise.

5. During the last 7 days, on how many days did you walk for at least 10 minutes at a time?

_____ days per week

No walking Skip to question 7

6. How much time did you usually spend walking each day on one of those days?

The average number of _____ hours _____ minutes per day

The last question is about the time you spent sitting on weekdays during the last 7 days. Include time spent at work, at home, while doing course work and during leisure time. This may include time spent sitting at a desk, visiting friends, reading, or sitting or lying down to watch television.

7. During the last 7 days, how much time did you spend sitting on a week day?

The average number of _____ hours _____ minutes per day

**References**

1. Craig CL, Marshall AL, Sjostrom M, Bauman A. International physical activity questionnaire: 12-country reliability and validity. Medicine and Science in Sports and Exercise. 2003;35:1381-95. https://doi.org/10.1249/01.MSS.0000078924.61453.FB

2. Macfarlane DJ, Lee CCY, Ho EYK, Chan KL, Chan DTS. Reliability and validity of the Chinese version of IPAQ (short, last 7 days). Journal of Science and Medicine in Sport. 2007;10:45-51. https://doi.org/10.1016/j.jsams.2006.05.003
