# Supplementary material for: Association between physical activity and falls among older adults in rural China: are there gender and age related differences?
Source: BMC Public Health. 2022 Feb 19;22:356. doi: 10.1186/s12889-022-12773-1 (PMC8858519; doi:10.1186/s12889-022-12773-1)
Supplement: Supplementary file 2 — Additional file 2: Appendix 2. Curvilinear regression. [file 12889_2022_12773_MOESM2_ESM.doc]

**Appendix 2: Curvilinear regression**

Previous studies found that low or high levels of PA were associated with falls in older adults [1, 2], indicating that there was a U-shaped relationship between PA and falls. We tested the curve relationship between PA and falls using curvilinear regression and test the trend in our study participants. Initially, we combined box plots and scatter plots to remove 3 samples of outliers, and finally included 3239 samples. Firstly, we made a scatter plot and added a fitting line for PA, MVPA and falls, and the result is shown in Figure S1. We found that there may be a quadratic nonlinear relationship between PA and falls. Secondly, quadratic regression prediction analysis was used to explore the relationship between PA and falls. Figure S2 presents this results.

**References**

1. Gill TM, Pahor M, Guralnik JM, Mcdermott MM, King AC, Buford TW, et al. Effect of structured physical activity on prevention of serious fall injuries in adults aged 70-89: randomized clinical trial (LIFE Study). BMJ. 2016;352:i245. https://doi.org/10.1136/bmj.i245

2. Pereira CLN, Baptista F, Infante P. Role of physical activity in the occurrence of falls and fall-related injuries in community-dwelling adults over 50 years old. Disabil Rehabil. 2014;36:117-24. https://doi.org/10.3109/09638288.2013.782355


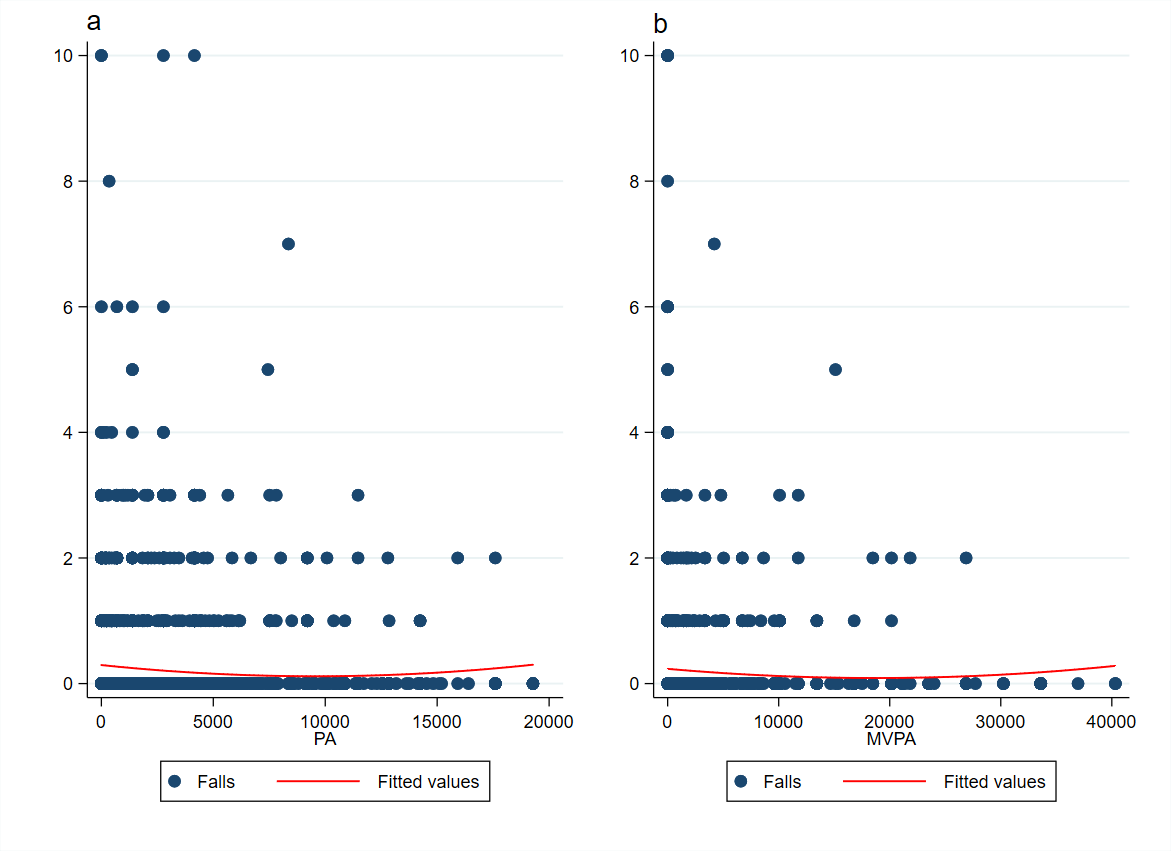


**Figure S1** A scatter plot of PA, MVPA and Falls (N=3,239). PA, physical activity; MVPA, moderate-to-vigorous physical activity


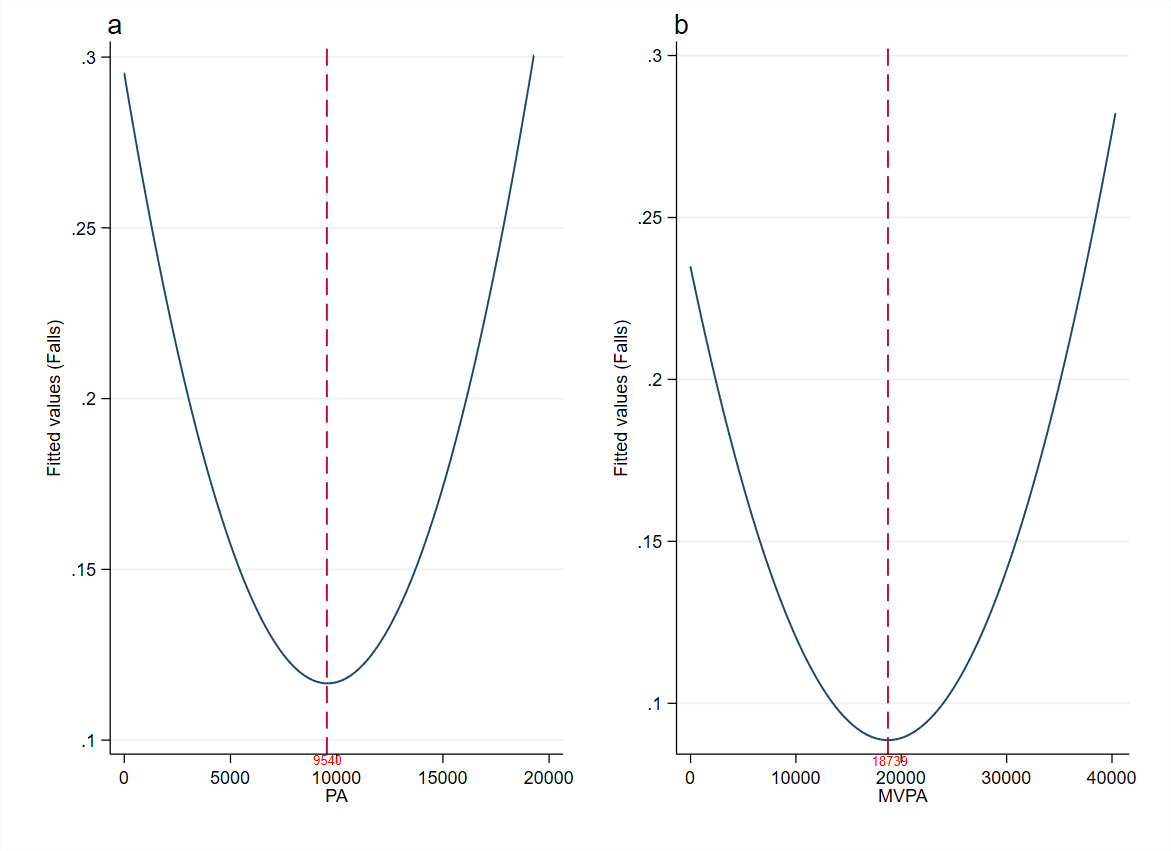


**Figure S2** Quadratic regression prediction between PA, MVPA and falls among older adults (N=3,239)
