# Supplementary figures and images for: Association between physical activity and falls among older adults in rural China: are there gender and age related differences?
Source: BMC Public Health. 2022 Feb 19;22:356. doi: 10.1186/s12889-022-12773-1 (PMC8858519; doi:10.1186/s12889-022-12773-1)

**Appendix 3: Distribution of falls**


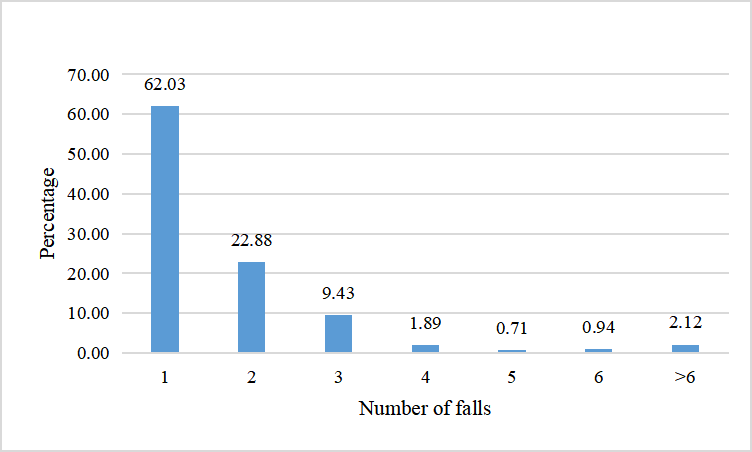


**Figure S3** Distribution of falls among older adults (N=3,242)

Supplement: Supplementary file 3 — Additional file 3: Appendix 3. Distribution of falls. [file 12889_2022_12773_MOESM3_ESM.doc]
