# Supplementary material for: Association between physical activity and falls among older adults in rural China: are there gender and age related differences?
Source: BMC Public Health. 2022 Feb 19;22:356. doi: 10.1186/s12889-022-12773-1 (PMC8858519; doi:10.1186/s12889-022-12773-1)
Supplement: Supplementary file 4 — Additional file 4: Appendix 4. Regression results based on single and multiple falls outcomes. [file 12889_2022_12773_MOESM4_ESM.doc]

**Appendix 4: Regression results based on single and multiple falls outcomes**

Falls status was classified as no falls (n=2,818), single fall (n=263), and multiple falls (n=161). Among older men, 1,064 did not fall, 76 had single fall and 41 had multiple falls. Among older women, 1,754 did not fall, 187 had single fall, and 120 had multiple falls. Among older adults younger than 75, 2,185 did not fall, 192 for single fall, and 122 for multiple falls. Among people 75 and older, 633 did not fall, 71 for single fall, and 39 for multiple falls. The results of multivariate regression are shown in Table S1, Table S2 and Table S3. The results of the number of falls as the continuous variable are shown in Table S4.

**Table S1 Associations between PA and MVPA with falls among older adults (N=3242)**

| **Variable** | **Single fall (n=263)** | | |  | **Multiple falls (n=161)** | | |
| --- | --- | --- | --- | --- | --- | --- | --- |
|  | **RRR** | **95% CI** | ***p-*valve** |  | **RRR** | **95% CI** | ***p-*valve** |
| **PA** |  |  |  |  |  |  |  |
| Low level | 1.00 |  |  |  | 1.00 |  |  |
| Moderate level | 0.89 | 0.65-1.22 | 0.471 |  | 0.93 | 0.63-1.39 | 0.735 |
| Elevated level | 0.64 | 0.44-0.94 | **0.021** |  | 0.55 | 0.33-0.92 | **0.022** |
| High level | 0.67 | 0.45-1.00 | 0.052 |  | 0.61 | 0.36-1.04 | 0.069 |
| **MVPA** |  |  |  |  |  |  |  |
| Low level | 1.00 |  |  |  | 1.00 |  |  |
| Moderate level | 1.84 | 0.88-3.85 | 0.104 |  | 1.57 | 0.59-4.17 | 0.363 |
| Elevated level | 0.87 | 0.51-1.46 | 0.587 |  | 0.82 | 0.42-1.62 | 0.573 |
| High level | 0.70 | 0.48-1.03 | 0.073 |  | 0.65 | 0.38-1.10 | 0.109 |

PA, physical activity; MVPA, moderate-to-vigorous physical activity; OR, odds ratio; CI, confidence interval. Models was adjusted for age, gender, education, household income, alcohol consumption, BMI, living alone, number of chronic disease, ADL score, PD score and MMSE score.

Table S2 Associations between PA and MVPA with falls stratified by gender

| **Variable** | **Male (n=1,181)** | | | | | | |  | **Female (n=2,061)** | | | | | | |
| --- | --- | --- | --- | --- | --- | --- | --- | --- | --- | --- | --- | --- | --- | --- | --- |
|  | **Single fall (n=76)** | | |  | **Multiple falls (n=41)** | | |  | **Single fall (n=187)** | | |  | **Multiple falls (n=120)** | | |
|  | **RRR** | **95% CI** | ***p-*valve** |  | **RRR** | **95% CI** | ***p-*valve** |  | **RRR** | **95% CI** | ***p-*valve** |  | **RRR** | **95% CI** | ***p-*valve** |
| **PA** |  |  |  |  |  |  |  |  |  |  |  |  |  |  |  |
| Low level | 1.00 |  |  |  | 1.00 |  |  |  | 1.00 |  |  |  | 1.00 |  |  |
| Moderate level | 1.26 | 0.68-2.35 | 0.465 |  | 0.87 | 0.38-2.01 | 0.750 |  | 0.82 | 0.56-1.19 | 0.286 |  | 0.99 | 0.62-1.56 | 0.951 |
| Elevated level | 0.71 | 0.33-1.54 | 0.386 |  | 0.61 | 0.22-1.68 | 0.337 |  | 0.63 | 0.40-0.98 | **0.042** |  | 0.53 | 0.29-0.96 | **0.037** |
| High level | 0.76 | 0.36-1.58 | 0.459 |  | 0.35 | 0.11-1.14 | 0.082 |  | 0.66 | 0.41-1.08 | 0.101 |  | 0.74 | 0.40-1.35 | 0.324 |
| **MVPA** |  |  |  |  |  |  |  |  |  |  |  |  |  |  |  |
| Low level | 1.00 |  |  |  | 1.00 |  |  |  | 1.00 |  |  |  | 1.00 |  |  |
| Moderate level | 6.50 | 2.08-20.35 | **0.001** |  | 2.68 | 0.31-23.31 | 0.372 |  | 1.02 | 0.36-2.94 | 0.966 |  | 1.45 | 0.48-4.37 | 0.504 |
| Elevated level | 0.78 | 0.27-1.36 | 0.644 |  | 0.64 | 0.13-3.09 | 0.583 |  | 0.90 | 0.49-1.64 | 0.724 |  | 0.87 | 0.40-1.87 | 0.719 |
| High level | 0.59 | 0.29-01.21 | 0.150 |  | 0.43 | 0.14-1.29 | 0.130 |  | 0.79 | 0.50-1.26 | 0.329 |  | 0.77 | 0.42-1.41 | 0.396 |

PA, physical activity; MVPA, moderate-to-vigorous physical activity; OR, odds ratio; CI, confidence interval. Models was adjusted for age, education, household income, alcohol consumption, BMI, living alone, number of chronic disease, ADL score, PD score and MMSE score.

Table S3 Associations between PA and MVPA with falls stratified by age

| **Variable** | **Aged 60-74 (n=2,499)** | | | | | | |  | **Aged 75-100 (n=743)** | | | | | | |
| --- | --- | --- | --- | --- | --- | --- | --- | --- | --- | --- | --- | --- | --- | --- | --- |
|  | **Single fall** **(n=192)** | | |  | **Multiple falls (n=122)** | | |  | **Single fall (n=71)** | | |  | **Multiple falls (n=39)** | | |
|  | **RRR** | **95% CI** | ***p-*valve** |  | **RRR** | **95% CI** | ***p-*valve** |  | **RRR** | **95% CI** | ***p-*valve** |  | **RRR** | **95% CI** | ***p-*valve** |
| **PA** |  |  |  |  |  |  |  |  |  |  |  |  |  |  |  |
| Low level | 1.00 |  |  |  | 1.00 |  |  |  | 1.00 |  |  |  | 1.00 |  |  |
| Moderate level | 0.86 | 0.58-1.26 | 0.429 |  | 0.87 | 0.55-1.39 | 0.560 |  | 1.01 | 0.57-1.79 | 0.975 |  | 1.15 | 0.52-2.54 | 0.735 |
| Elevated level | 0.68 | 0.44-1.06 | 0.089 |  | 0.32 | 0.16-0.64 | **0.001** |  | 0.53 | 0.24-1.17 | 0.116 |  | 1.50 | 0.64-3.55 | 0.354 |
| High level | 0.71 | 0.46-1.10 | 0.127 |  | 0.66 | 0.38-1.15 | 0.141 |  | 0.56 | 0.20-1.56 | 0.267 |  | - | - | 0.989 |
| **MVPA** |  |  |  |  |  |  |  |  |  |  |  |  |  |  |  |
| Low level | 1.00 |  |  |  | 1.00 |  |  |  | 1.00 |  |  |  | 1.00 |  |  |
| Moderate level | 2.36 | 1.10-5.06 | **0.027** |  | 1.53 | 0.51-4.57 | 0.450 |  | - | - | 0.983 |  | 1.72 | 0.19-15.83 | 0.631 |
| Elevated level | 1.01 | 0.58-1.78 | 0.967 |  | 0.89 | 0.41-1.90 | 0.754 |  | 0.46 | 0.11-2.01 | 0.302 |  | 0.67 | 0.15-3.01 | 0.598 |
| High level | 0.76 | 0.50-1.16 | 0.206 |  | 0.72 | 0.41-1.25 | 0.237 |  | 0.49 | 0.16-1.43 | 0.191 |  | 0.28 | 0.04-2.18 | 0.225 |

PA, physical activity; MVPA, moderate-to-vigorous physical activity; OR, odds ratio; CI, confidence interval. Models was adjusted for gender, education, household income, alcohol consumption, BMI, living alone, number of chronic disease, ADL score, PD score and MMSE score.

**Table S4** Association between between PA and MVPA with falls

|  | **All** | | |  | **Male** | | | |  | **Female** | | |  | **Aged 60-74** | | |  | **Aged 75-100** | | |
| --- | --- | --- | --- | --- | --- | --- | --- | --- | --- | --- | --- | --- | --- | --- | --- | --- | --- | --- | --- | --- |
|  | **B** | **SE** | ***p*-value** |  | **B** | **SE** | | ***p*-value** |  | **B** | **SE** | ***p*-value** |  | **B** | **SE** | ***p*-value** |  | **B** | **SE** | ***p*-value** |
| **PA** |  |  |  |  |  |  | |  |  |  |  |  |  |  |  |  |  |  |  |  |
| Low level | 1.000 |  |  |  | 1.000 |  | |  |  | 1.000 |  |  |  | 1.000 |  |  |  | 1.000 |  |  |
| Moderate level | -0.008 | 0.033 | 0.810 |  | 0.053 | | 0.056 | 0.343 |  | -0.032 | 0.041 | 0.428 |  | -0.015 | 0.040 | 0.710 |  | 0.021 | 0.054 | 0.695 |
| Elevated level | -0.093 | 0.036 | **0.010** |  | -0.027 | | 0.060 | 0.649 |  | -0.133 | 0.045 | **0.003** |  | -0.117 | 0.043 | **0.007** |  | -0.012 | 0.062 | 0.841 |
| High level | -0.079 | 0.037 | 0.034 |  | -0.034 | | 0.057 | 0.551 |  | 0.098 | 0.049 | **0.048** |  | -0.071 | 0.043 | 0.100 |  | -0.140 | 0.079 | 0.075 |
| **MVPA** |  |  |  |  |  | |  |  |  |  |  |  |  |  |  |  |  |  |  |  |
| Low level | 1.000 |  |  |  | 1.000 | |  |  |  | 1.000 |  |  |  | 1.000 |  |  |  | 1.000 |  |  |
| Moderate level | 0.082 | 0.089 | 0.361 |  | 0.229 | | 0.153 | 0.135 |  | 0.023 | 0.109 | 0.835 |  | 0.102 | 0.102 | 0.318 |  | -0.010 | 0.185 | 0.957 |
| Elevated level | -0.061 | 0.049 | 0.218 |  | -0.051 | | 0.080 | 0.525 |  | -0.063 | 0.062 | 0.309 |  | -0.052 | 0.056 | 0.360 |  | -0.091 | 0.100 | 0.360 |
| High level | -0.063 | 0.033 | 0.060 |  | -0.049 | | 0.048 | 0.310 |  | -0.063 | 0.045 | 0.169 |  | -0.049 | 0.037 | 0.190 |  | -0.119 | 0.077 | 0.119 |

PA, physical activity; MVPA, moderate-to-vigorous physical activity; B, beta, SE, standard error. Models was adjusted for gender, education, household income, alcohol consumption, BMI, living alone, number of chronic disease, ADL score, PD score and MMSE score.
